# Associative Memory Impairments Are Associated With Functional Alterations Within the Memory Network in Schizophrenia Patients and Their Unaffected First-Degree Relatives: An fMRI Study

**DOI:** 10.3389/fpsyt.2019.00033

**Published:** 2019-02-19

**Authors:** Viola Oertel, Dominik Kraft, Gilberto Alves, Christian Knöchel, Denisa Ghinea, Helena Storchak, Silke Matura, David Prvulovic, Robert A. Bittner, David E. J. Linden, Andreas Reif, Michael Stäblein

**Affiliations:** ^1^Laboratory for Neuroimaging, Department of Psychiatry, Psychosomatic Medicine and Psychotherapy, Goethe University, Frankfurt am Main, Germany; ^2^Brain Imaging Centre, Goethe University, Frankfurt am Main, Germany; ^3^Post Graduation in Psychiatry and Mental Health, Federal University of Rio de Janeiro, Rio de Janeiro, Brazil; ^4^MRC Centre for Neuropsychiatric Genetics and Genomics, School of Medicine, Institute of Psychological Medicine and Clinical Neurosciences, Cardiff University, Cardiff, United Kingdom

**Keywords:** face-name association task, associative memory, schizophrenia, schizophrenia spectrum, fMRI

## Abstract

Memory impairments are a major characteristic of schizophrenia (SZ). In the current study, we used an associative memory task to test the hypothesis that SZ patients and first-degree relatives have altered functional patterns in comparison to healthy controls. We analyzed the fMRI activation pattern during the presentation of a face-name task in 27 SZ patients, 23 first-degree relatives, and 27 healthy controls. In addition, we performed correlation analyses between individual psychopathology, accuracy and reaction time of the task and the beta scores of the functional brain activations. We observed a lower response accuracy and increased reaction time during the retrieval of face-name pairs in SZ patients compared with controls. Deficient performance was accompanied by abnormal functional activation patterns predominantly in DMN regions during encoding and retrieval. No significant correlation between individual psychopathology and neuronal activation during encoding or retrieval of face-name pairs was observed. Findings of first-degree relatives indicated slightly different functional pattern within brain networks in contrast to controls without significant differences in the behavioral task. Both the accuracy of memory performance as well as the functional activation pattern during retrieval revealed alterations in SZ patients, and, to a lesser degree, in relatives. The results are of potential relevance for integration within a comprehensive model of memory function in SZ. The development of a neurophysiological model of cognition in psychosis may help to clarify and improve therapeutic options to improve memory and functioning in the illness.

## Introduction

Schizophrenia (SZ) is a severe mental disease, with patients not only suffering from “positive” (e.g., delusions, hallucinations, disturbances of thoughts) and “negative” symptoms (e.g., loss of energy, flattened affect) (1), but also from various cognitive deficits. For instance, associative memory deficits are commonly observed in SZ (2–6). The underlying functional network of associative memory processes includes the prefrontal cortex (PFC), the hippocampus (HC), the medial temporal cortex (MTL), the parahippocampal and fusiform gyrus, as well as other cerebral structures (parietal-temporal-occipital association cortex, cerebellum, cingular cortex, thalamus) (7–11). The formation of complex cross-modal associations, such as face–name pairs, is mainly related to the HC (12). According to Sperling et al. (13) and Kirwan and Stark (14), activation of the anterior HC is particularly closely associated with successful memory encoding.

According to a meta-analysis by Achim and Lepage (2). during encoding, schizophrenia patients showed decreased activation of the left inferior PFC, the right middle frontal gyrus, the right medial frontal gyrus, and the right posterior HC. During retrieval, they identified lower activation in SZ compared with controls in several frontal regions, in the right subgenual region, in the thalamus bilaterally, in the left anterior HC, in the right fusiform gyrus and in the cerebellum bilaterally. In contrast, the authors identified higher functional activation in the right anterior MTL in SZ patients compared to controls.

There is also evidence of subtle memory impairments in first-degree relatives of SZ patients (15–17). Stolz et al. (18) reported the intermediate performance of relatives—between SZ patients and controls—in associative memory performance. This was in line with their fMRI findings, indicating no differences between relatives and controls in the functional activation pattern during encoding, but a difference in the PFC, the thalamus and the insula during retrieval in the relatives group compared to controls. Di Giorgio et al. (19) observed hippocampus-parahippocampal abnormalities during the encoding of a memory task in SZ patients and relatives compared to the controls. Pirnia et al. (16) used a face-name associative memory task and a region-of-interest (ROI)-analysis of HC and MTL to explore the fMRI pattern during successful vs. unsuccessful encoding in SZ patients, first-degree relatives and healthy controls. They observed similar hippocampal hypo-activations during successful vs. unsuccessful encoding in SZ patients and their unaffected relatives, although hippocampal volume reductions and hyper-activations in temporo-occipital and parietal regions were restricted to the patient group.

In summary, the few studies which exist show inconsistent results that elucidate the importance of the investigation of patients as well as first-degree relatives with regard to their memory performance and underlying functional activation patterns. This line of research is important because it helps clarify neural systems underlying cognitive deficits in schizophrenia and potential endophenotypes, which is crucial for an integration of associative memory paradigms in translational research and the development of new cognitive markers of disease progression and treatment effects. We tested patients with SZ, first degree relatives, and controls without a family history of schizophrenia with an associative memory paradigm during fMRI. We expected impaired performance and recruitment of memory-relevant brain regions in the patient compared to relatives and controls, but also more subtle impairments in the relatives group.

## Methods and Materials

### Participants

We included 27 healthy control subjects (CON) {*M*_age_(mean) = 34.22 years (SD[standard deviation] = ±11.38)}, 27 patients (SZ) (*M*_age_ = 37.22 years [*SD* = ±9.14]) with the diagnosis of SZ according to DSM IV (20) and 23 first-degree relatives of SZ patients with no history of psychiatric disorders (REL) (*M*_age_ = 43.56 years [*SD* = ±14.25]). All imaging data were controlled for any neuroanatomical abnormality. The subsamples were matched for age, gender, and years of education (see Table 1 for details). Only right-handed [EHI; (21)] subjects were included.

**Table 1:** Group comparisons of sociodemographic and cognitive data across groups (corrected for multiple comparisons using the Bonferroni correction).

	**SZ *M (SD)***	**REL *M (SD)***	**CON *M(SD)***	**Significance F*_**(*df*)**_***
Number	27	23	27	
Gender *(f/m)*	9/20	19/5	17/13	*χ2* = 0.57, *p* = 0.44
Age *(years)*	37.22 (9.14)	43.56 (14.25)	34.22 (11.38)	*F*_(75)_ = 1.96, *p* = 0.14
Education *(years)*	14.94 (3.11)	15.63 (2.31)	16.55 (1.75)	*F*_(75)_ = 2.93, *p* = 0.06
Education mother *(years)*	13.09 (2.59)	13.20 (3.89)	16.71 (1.54)	*F*_(75)_ = 2.05, *p* = 0.14
Education father *(years)*	14.13 (2.69)	13.42 (3.34)	15.68 (1.38)	*F*_(75)_ = 2.91, *p* = 0.06
RHS *(points)*	33.92 (7.88)	26.53 (4.92)	23.85 (3.67)	*F*_(75)_ = 18.57, *p* < 0.001** SZ/CON, *p* < 0.001** SZ/REL, *p* < 0.001**
PANSS *(only patients)*	Pos: 17.08 (4.85), Neg: 16.24 (6.09), Gen: 32.32 (7.38), Total: 65.64 (15.22)			
MWT-B *(t-score)*	51.80 (9.51)	58.62 (10.82)	62.50 (8.18)	*F*_(75)_ = 8.60, *p* < 0.001** SZ/CON, *p* = 0.01* REL/CON, *p* = 0.03*
TMT A *(t-score)*	40.92 (13.99)	49.19 (10.96)	47.00 (8.89)	*F*_(75)_ = 2.42, *p* = 0.09
Associative memory	Time (IR–DR): *F*_(73)_ = 43.41, *p* < 0.001** group: *F*_(73)_ = 10.65, *p* < 0.001** Interaction group*IR–DR: *F*_(73)_ = 0.44, *p* = 0.647			
IR *(points)*	18.54 (5.32)	23.80 (4.53)	23.46 (4.68)	*t*_(75)_ = 6.24, *p* = 0.001** SZ/CON *p* = 0.004** SZ/REL *p* = 0.004**
DR *(points)*	14.95 (6.16)	21.30 (5.40)	20.28 (6.43)	*t*_(75)_ = 5.03, *p* = 0.003** SZ/CON *p* = 0.01* SZ/REL *p* = 0.006**
RT *(ms)*	4568.59 (703.86)	4103.72 (696.79)	4013.62 (725.18)	*t*_(75)_ = 2.96, *p* = 0.03* SZ/CON, *p* = 0.02*

*SZ, SZ patients; REL, relatives; CON, controls; M, mean; SD, standard deviation; RHS, Revised Hallucination Scale; MWT-B, Multiple-Choice-Word-Comprehension-Test; TMT, Trail-Making Test; IR, associative memory immediate retrieval; DR, associative memory delayed retrieval (post-scanning); RT, associative memory reaction time*.

* *p < 0.05*,

** *p < 0.01. MWT-B scores were included as covariates into the associative memory analyses*.

To verify the diagnosis or exclude possible psychiatric disorders, the German version of the Structured Clinical Interview (SCID-I and –II) for DSM-IV (22) was applied. Revised Hallucinations Scale [RHS; (23)] was used to screen for hallucinatory predisposition. Premorbid intelligence was measured by the German version of the Multiple-Choice-Word-Comprehension Test [MWT-B; (24)] and psychomotor speed was measured with the Trail-Making-Test A [TMT A; (25)]. All subjects were caucasian. They provided written informed consent according to experimental procedures approved by the ethical board of the medical school of the Goethe-University, Frankfurt, Germany.

Patients were under current treatment at the Department of Psychiatry, Goethe-University, Frankfurt, Germany, and in a non-acute, stable clinical condition (see Table 1). None of them had any comorbid axis-I or -II disorders according to the DSM-IV criteria (20) or current drug abuse during the last three months preceding the study. We assessed the duration of illness, age of onset and any psychiatric medication taken. The current extent of psychopathological symptoms in patients was assessed using the German version of the Positive and Negative Syndrome Scale (PANSS; (26)). We ensured that SZ patients did not fulfill the criteria for severe acute symptoms in the PANSS (all scores < 85 points) (27). A stable psychopharmacological medication (for at least 4 weeks prior to assessment date) and not receiving benzodiazepine for a month were necessary for patients to be enrolled. All patients were currently treated with antipsychotics either in monotherapy or in combination with other antipsychotics. Antipsychotic medication doses were converted into chlorpromazine equivalents (28) for further analyses (see Table S1).

### Experimental Procedure

All subjects underwent functional and structural imaging at the Frankfurt University Brain Imaging Center, Frankfurt, Germany. MR images were acquired using a Trio 3-T scanner (Siemens Medical Systems, Erlangen, Germany), with a standard head coil for radiofrequency transmission and signal reception.

**Figure 1:**
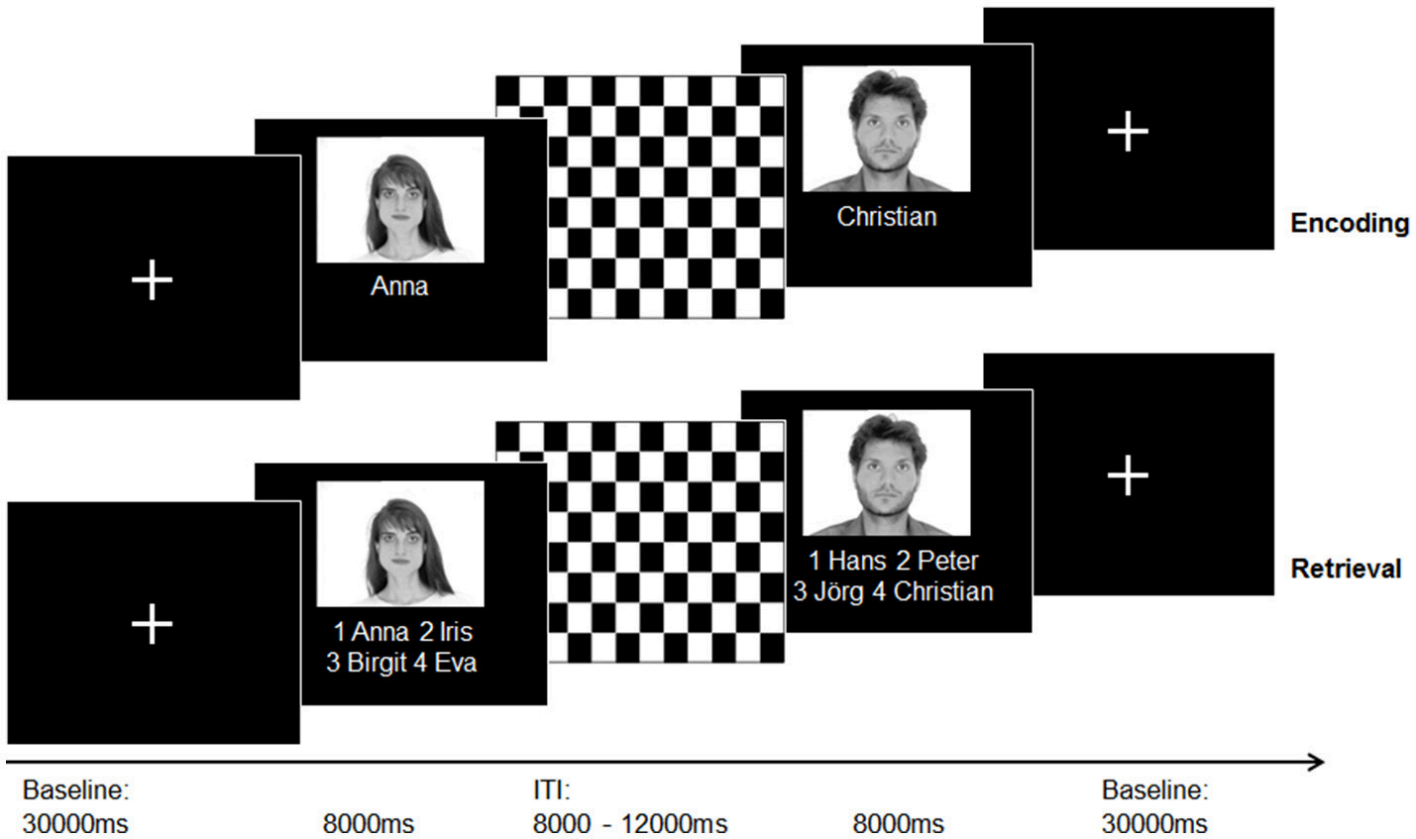
Illustration of the experimental paradigm (face-name-association task) according to Sperling et al. (11) during the acquisition of an fMRI sequence. A total of 30 photographs of emotionally neutral, gray-scaled faces taken from the “AR” face database (30) were randomly assigned to 30 popular German forenames taken from an online database with popular German names (www.beliebtevornamen.de).

For T1-weighted structural imaging, an optimized 3D modified driven equilibrium Fourier transform sequence [3D MDEFT; 176 slices, 1.0mm slice thickness; (29)] was applied. During the acquisition of three functional runs (T2^*^ weighted Echo-Planar-Imaging (EPI) sequence, a face–name association paradigm developed by Sperling et al. (11) was presented. All stimuli were presented and answers logged via the Presentation Software (Version 10.3 Neurobehavorial Systems Inc.). Stimuli were projected on a frosted screen using a projector, which was visible for all subjects via a mirror mounted on top of the head coil inside the scanner. See Figure 1 for an illustration of the experimental procedure and Supplementary Material for a detailed task and sequences description.

Thirty minutes after MRI scans, subjects underwent post-scanning face–name retrieval. Participants received a questionnaire with the same face–name pairs (with three distractor names) and were instructed to mark the correct names. This task was introduced to assess delayed recall memory functioning. We created a self-constructed questionnaire to explore memory strategies at the end of assessment. The participants were asked whether they used the following potential memory strategies to remember the items: pronouncing names in a low voice, visualization, recollection of striking features, remembering the names by constructing a story, and association of the faces/names with known persons (answer: yes/no). In addition, participants were also asked to rate their attention and concentration during the scan on a 5-point Likert scale (0 = low, 5 = high).

### Statistical Analysis

#### Neuropsychological and Clinical Data

All cognitive and clinical test results were analyzed using SPSS® 22.0 (Statistical Package for Social Sciences, SPSS Inc., USA). After differentiating between parametric and non-parametric data by applying the Kolmogorov-Smirnov test, appropriate statistical tests were conducted. Bonferroni correction (α = 0.05) was applied to correct for multiple comparisons. We performed group comparisons (ANOVAs) with group being a fixed factor with three levels (CON, REL, SZ) and the test scores of the cognitive and clinical tests (TMT A, MWT-B, RHS) as dependent variables.

#### Associative Memory Performance

Regarding the face-name-association paradigm, the mean accuracy of immediate retrieval (IR), delayed retrieval (DR; post-scanning) and the overall mean reaction time (RT) during the immediate retrieval of each participant was computed. We performed group comparisons with repeated measures ANOVA with IR, DR and group being fixed factors with three levels (CON, REL, SZ). We also computed an ANOVA with RT as a dependent variable and group as a fixed factor. Memory strategies were analyzed using adequate statistical tests to compare results between groups (see Table S2).

#### Imaging Data

For (f)MRI data a standard preprocessing pipeline was applied (see Supplementary Material). Two general linear models (GLM) were computed separately for encoding and retrieval, with each containing 230 time courses (77 participants × 3 runs; we excluded 1 run due to no correct logged responses). Successful or unsuccessful encoding was defined as hits or misses in the respective retrieval trials. The GLM for encoding included two task phases/conditions as separate predictors (successful encoding, ITI) and seven confounding predictors (six z-transformed motion parameters obtained during fMRI preprocessing, unsuccessful encoding). The GLM for the retrieval run also included two predictors (successful retrieval, ITI) and the respective confounders. Since the majority of participants did not make any mistakes during retrieval, we added the “unsuccessful” predictor as a confounder to maximize the explained variability. Event-related fMRI activity was modeled by convolving the predictors with a canonical hemodynamic response function (HFR). In the first level of random effect analysis, condition effects for each subject (beta-values) were estimated.

Obtained beta-values were used to calculate statistical comparisons (F-statistics) between experimental conditions (encoding, ITI; retrieval, ITI). Activations associated with successful encoding (successful encoding>ITI) and successful retrieval (successful retrieval>ITI) were computed for the whole sample using linear contrasts (*t*-statistics). To correct for multiple comparisons, FDR correction (31) with a threshold of *p* < 0.001 (minimum cluster size > 100 mm^3^) was applied.

Random effects analysis was conducted to test for differences in activation between groups (ANOVA). Planned comparisons between groups were conducted within memory conditions (encoding, retrieval), resulting in three between group comparisons each. For the group comparisons, an initial voxel level threshold was set to *p* = 0.001 uncorrected. To correct for multiple comparisons, the Cluster Threshold Estimator Plugin (Monte Carlo Simulation: 1,000 iterations, *p* < 0.05) implemented in BrainVoyager QX 2.8 (Brain Innovation Maastricht, the Netherlands) was applied.

Furthermore, we computed regions-of-interest (ROI)-analyses of anatomically defined brain regions: bilateral prefrontal cortices (PFC), bilateral hippocampus (HC) and bilateral medial temporal lobe (MTL). Activation patterns of ROIs were thresholded at an initial level of *p* < 0.05 uncorrected, cluster-level corrected (Monte Carlo Simulation: 1,000 iterations, *p* < 0.05). The anatomically defined regions were based on the automated anatomical labeling atlas in WFU PickAtlas v2.0 (32) and included the following clusters: {hippocampus:; PFC: −40, 20, 22 [3,583 voxels]; 41, 10, 33 (3393); see Figure S1 for a ROI mask}.

#### Correlation Analysis

Correlation analyses were performed to investigate the relationship of between-group differences with clinical and cognitive variables, all corrected for multiple comparisons using Bonferroni correction. Clusters displaying significant between-group differences during encoding and retrieval were targeted for beta-value extraction from spheres with a 44 mm radius around the peak voxel using the BrainVoyager VOI function. These beta values were correlated using bivariate correlation analyses (Spearman product-moment correlation or Pearson correlation coefficient, two-tailed) with associative memory performance (IR, DR, RT) and clinical scores (RHS) for each group individually. In the patient group, we controlled for the potential influence of medication performing bivariate correlation analyses (Spearman product-moment correlation, two-tailed) between the beta scores of significant regions and medication dosage using chlorpromazine equivalents. Accordingly, correlation analyses between the performance in the face-name task (IR, DR, RT) and acute symptomatology (PANSS) were conducted.

## Results

### Neuropsychological and Clinical Data

There were significant group differences in predisposition toward hallucinations (RHS), indicating higher values in the patient group compared to REL and CON [*F*_(75)_ = 18.57, *p* < 0.001], and slightly higher values in REL in contrast to CON without reaching statistical significance.

For crystallized intelligence (MWT-B) significant group differences [*F*_(75)_ = 8.60, *p* < 0.001] were observed, with significant differences between patients and controls (*p* = 0.01) and relatives and controls (*p* = 0.03). Due to group differences, we included MWT-B scores as a covariate into the following analyses. There was no difference in psychomotor speed (TMT A) between groups [*F*_(75)_ = 2.42, *ns*]. Effect sizes calculation (Cohens d) indicated for TMT A an effect size of *d* = 0.64 and for MWT-B *d* = 1.29.

### Associative Memory Performance

For associative memory a significant effect of time [immediate vs. delayed; *F*_(73)_ = 43.40, *p* < 0.001] and group [*F*_(73)_ = 10.65, *p* < 0.001] was observed, but no interaction group^*^IR–DR [*F*_(73)_ = 0.44, *p* = 0.647].

**Figure 2:**
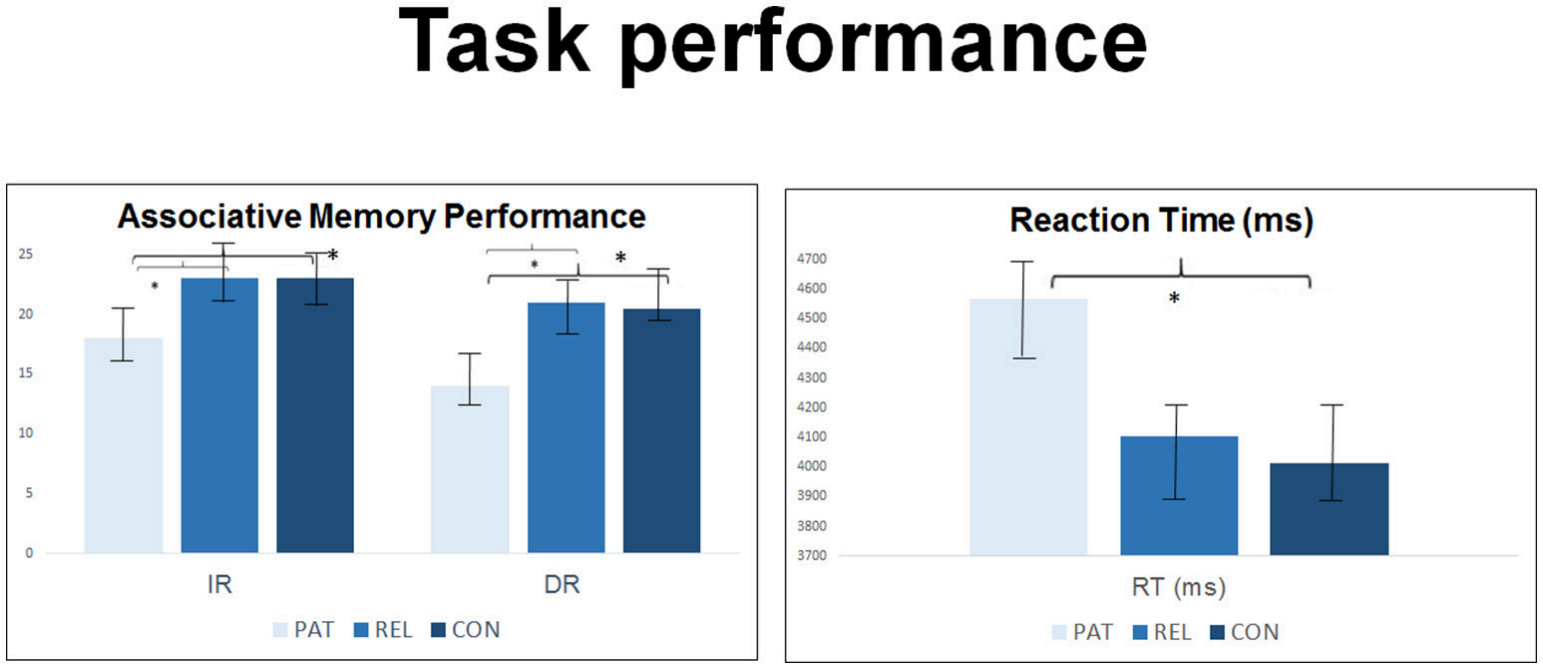
Group comparison in *n* = 27 controls, *n* = 23 first-degree relatives and *n* = 27 SZ patients regarding accuracy and reaction time of the face-name-association-task during the acquisition of an fMRI sequence and during post-scanning debriefing. *M*, mean; *SD*, standard deviation. *Indicates statistial significance.

We observed significant group effects in immediate [*t*_(75)_ = 6.24, *p* = 0.001] and in delayed [*t*_(75)_ = 5.03, *p* = 0.003] retrieval. Group differences in immediate retrieval were caused by significantly lower correct responses in SZ compared to REL and CON. A comparable pattern was displayed for delayed retrieval indicating differences in performance between SZ and CON and SZ and REL during the retrieval of face-name-pairs (all *p* < 0.05). SZ had significantly higher reaction time compared to CON [*t*_(75)_ = 2.96, *p* = 0.03]. REL showed intermediate values between SZ and CON without reaching statistical significance in *post-hoc* group contrasts (see Table 1 and Figure 2). Immediate retrieval had an effect size of *d* = 1.10, delayed retrieval an effect size of *d* = 1.03 and the reaction time had an effect size of *d* = 0.70.

The memory strategies, self-rated attention and concentration showed significant variance between groups (all *p* > 0.05; see Table S2).

### Imaging Results

#### Main Effect

During encoding, we observed the main effect of encoding vs. ITI in the right superior temporal gyrus, left cuneus, right inferior occipital gyrus, right caudate, left inferior frontal gyrus and left fusiform gyrus. The main effect of retrieval vs. ITI was detected in the left inferior parietal lobule, left inferior occipital gyrus, right precentral gyrus (more activated), right cuneus and left medial frontal gyrus (all *p* < 0.001, FDR corrected) (see Table 2).

**Table 2:** Main effect for successful encoding (>ITI) and successful retrieval (>ITI) for the whole sample using linear contrasts (*t*-statistics).

**Anatomical region**	**R/L**	**BA**	**Talairach coordinates**			**Cluster size**	***t*_**(76)**_**
			**x**	**y**	**z**	**(voxels/mm^3^)**	
**Encoding>ITI**							
Superior temporal gyrus	R	13	54	−40	19	4581	−7.3987
Inferior occipital gyrus	R	18	27	−88	−8	18442	10.8565
Cuneus	L	18	0	−79	7	95712	−16.4915
Caudate (Body)	R	*	15	−4	19	2946	7.7406
Inferior frontal Gyrus	L	47	−48	23	1	26134	8.1440
Fusiform gyrus	L	19	−30	−82	−14	18516	9.9319
**RETRIEVAL>ITI**							
Inferior parietal lobule	L	40	−42	−37	52	188875	13.1462
Inferior occipital gyrus	L	18	−27	−85	−14	95021	13.2546
Precentral gyrus	R	6	30	−13	64	2998	7.1636
Cuneus	R	18	9	−85	25	61462	−10.7883
Medial frontal gyrus	L	10	0	56	10	4274	−6.8900

To correct for multiple comparisons, FDR correction (31) with a threshold of p < 0.001 (minimum cluster size>100 mm^3^) was applied. R/L, Right/Left; BA, Brodmann area; *, no Brodmann area. Talairach coordinates, anatomical regions and Brodmann areas refer to peak voxel of cluster. PAT, SZ patients; REL, relatives; CON, controls.

#### Second Level Analyses: Between-Group Comparisons

##### Group contrast encoding

**Figure 3:**
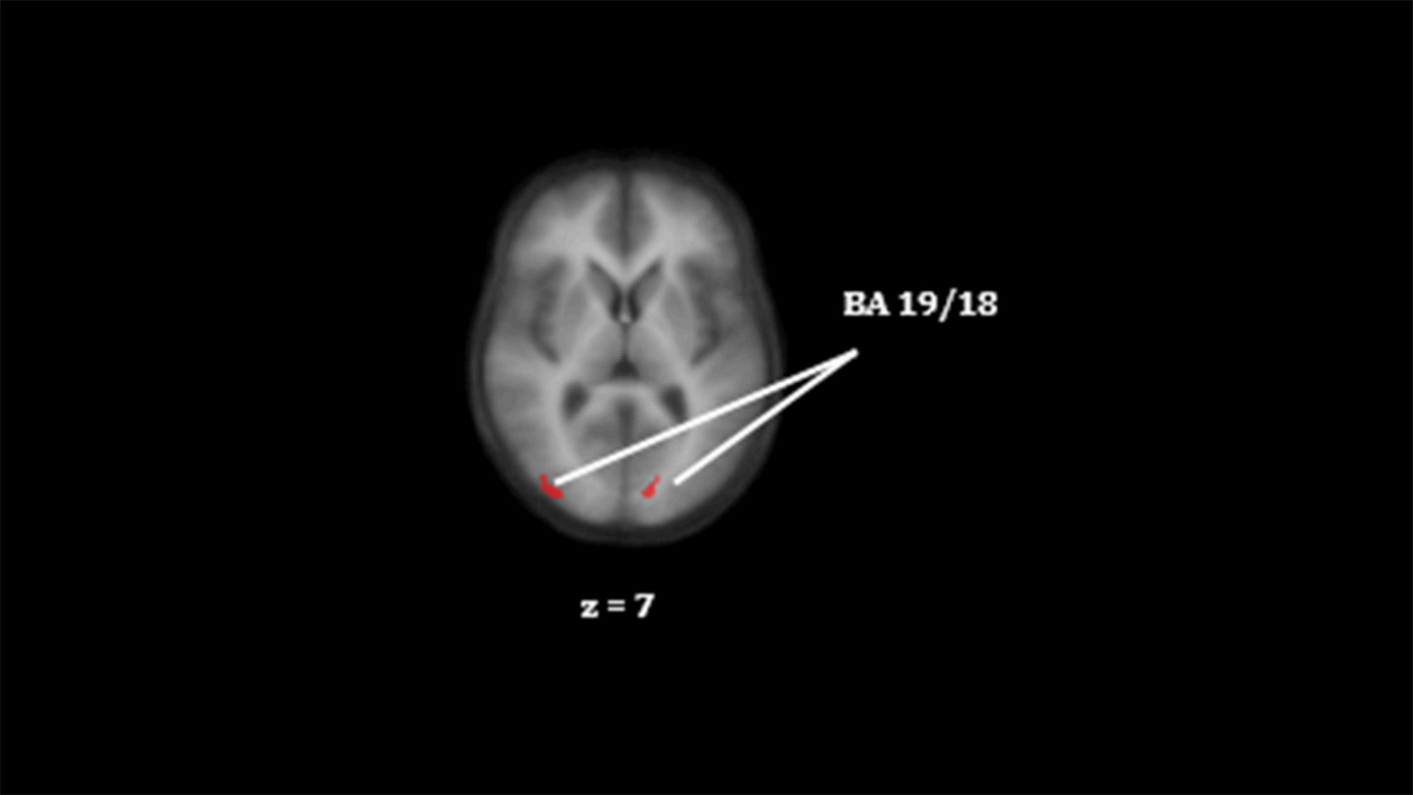
Differences in activation in the right middle occipital gyrus and the left lingual gyrus between CON and SZ (CON > SZ) for successful encoding. The color red indicate lower activation in the reference group. Colors do not represent statistical values and are for visual purposes only, for statistical information please see Table 3. Clusters on an anatomical image averaged over all participants in the Talairach standard space, according to the radiological convention. BA indicate the Brodmann area.

We observed significant lower activation in SZ compared to CON in right middle occipital gyrus, left lingual gyrus, left cuneus and right cingulate gyrus (see Table 3 and Figure 3). SZ showed significant lower activation compared to REL in right cingulate gyrus, left lingual gyrus, and left superior frontal gyrus. Significant lower activation in REL compared to CON were observed in the right inferior frontal gyrus and bilateral middle occipital gyrus.

**Table 3:** Statistical group comparisons of functional brain activation differences between groups for successful encoding and retrieval (>ITI).

**Anatomical region**	**R/L**	**BA**	**Talairach coordinates**			**Cluster Size**	***t*_**(76)**_**
			**x**	**y**	**z**	**(voxels/mm^3^)**	
**ENCODING**							
**CON>SZ**							
Middle occipital gyrus	R	19	33	−85	7	398	2.2548
Lingual gyrus	L	18	−15	−76	7	346	−3.2746
Cuneus	L	17	−16	−76	7	2110	−3.2971
Cingulate gyrus	R	24	25	−17	40	280	−1.728
**SZ>REL**							
Cingulate gyrus	R	24	24	−19	50	139	1.6457
Lingual gyrus	L	18	−15	−76	7	260	−3.2746
Superior frontal gyrus	L	6	−18	11	65	181	−1.7561
**CON>REL**							
Inferior frontal gyrus	R	9	51	17	22	130	−3.4613
Middle occipital gyrus	R	19	33	−85	13	559	2.7364
	L	19	−27	−80	10	109	1.6380
**RETRIEVAL**							
CON>SZ							
Cingulate gyrus	R	31	18	−37	30	184	2.1345

SZ, schizophrenia patients; REL, schizophrenia relatives; CON, controls; L, left; R, right; BA, Broadman area, ^*^, no Brodmann area; p < 0.05, corrected using cluster thresholding approach with initial single-voxel threshold of p < 0.001 (uncorrected); Talairach coordinates, anatomical regions and Brodmann areas refer to peak voxel of cluster.

##### Group contrast retrieval

SZ showed significantly lower activation compared to CON in the right cingulate gyrus). No significant group differences between CON and REL or between SZ and REL were found (see Table 3).

##### *Post-hoc* ROI analysis: hippocampus-related group contrasts

ROI analysis of HC brain activation during encoding (>ITI) revealed significant group differences in the left HC in all computed contrasts (SZ<CON, REL<CON, SZ>REL) and a significant contrast in the right HC between CON and SZ. Lower activations during retrieval were found in the HC bilaterally in SZ compared to CON, whereas higher activation was observed in the parahippocampal gyrus bilaterally (*p* < 0.05). REL showed compared to CON lower activation in left HC and higher activation in parahippocampal gyrus (all *p*'s < 0.05; see Table S3, Figure 3). We did not observe any differences between SZ and REL (*p* > 0.05).

##### Post-hoc ROI analysis: prefrontal gyrus-related group contrasts

During encoding (> ITI), we observed significantly lower left PFC activation in SZ compared to CON and REL. We observed bilateral PFC group differences during retrieval. During retrieval, REL and SZ presented significantly lower left and PFC activation in comparison to CON (all *p* < 0.05). REL and SZ showed no significant group contrasts.

##### Influence of associative memory performance

We computed additional covariate analyses, using the immediate retrieval, delayed retrieval and reaction time scores as covariates and the main imaging scores during encoding and retrieval as dependent variables. However, these analyses revealed no significant influence of cognitive performance on the imaging results (*p* > 0.05).

### Correlation Analyses

Across groups, immediate retrieval, delayed retrieval and reaction time was mutually associated (*r* = −0.376, *p* < 0.001). In CON, the higher the RHS scores, the lower the delayed retrieval performance (rho = −0.18, *p* = 0.03); and the lower the RHS scores, the lower the reaction time (rho = 0.42, *p* = 0.02). All other computed correlation analyses between clinical scores and cognitive performance or fMRI pattern did not show any significant differences between groups.

### Influence of Psychiatric Medication

None of the associative memory scores (IR, DR, RT), clinical scores (PANSS, RHS) or fMRI findings were significantly associated with chlorpromazine equivalents in the patient group (all *p* > 0.05).

## Discussion

SZ patients showed significantly lower accuracy in immediate recall (during fMRI) and delayed recall (after fMRI) of face-name-pairs compared to relatives and controls. This was accompanied by higher reaction times in patients compared to controls during immediate recall. Relatives showed slightly higher reaction times and slightly lower accuracy compared to controls. fMRI pattern indicated a network related to cognition (mainly DMN regions) and visual perception/(occipital lobe) to be active during the association of faces to names.

Our results confirm the previous findings which indicated deficits in SZ patients in various tasks exploring associative memory, including verbal and non-verbal associative tasks (6), tasks using free recall vs. recognition of memory items (33, 34), tasks involving different difficulty levels of processing (i.e., perception vs. categorization; (35) and tasks with trained vs. non-trained recall (36). Our results of slightly impaired associative memory in relatives supports the previous findings of subtle memory impairments in first-degree relatives (15–17).

One assumption is that SZ patients have deficits to use any memorization strategy if they are not directly instructed (37, 38). In the present study, we did not find any variance in the use of memory strategies or attention or concentration differences across groups during post scanning debriefing. However, none of the other clinical scores were significantly associated with cognitive performance or the fMRI pattern across groups. Therefore, task performance does not seem to be affected by these parameters or by medication in the patient group. Furthermore, we controlled for the potential influence of crystallized intelligence. Therefore, we postulate that impairments in associative memory in SZ patients are not directly related to illness state, psychiatric treatment or general intelligence.

The canonical memory network activated by the task confirms previous knowledge about functional patterns underlying associative memory tasks (7–9, 11, 39, 40); we observed functional activation in DMN regions (medial frontal gyrus, inferior parietal lobe) and in occipital lobe regions (cuneus, inferior occipital gyrus, fusiform gyrus) during the task.

Beside differences in the visual cortex, the pattern of differences between controls and SZ patients included parts of the DMN during encoding and during retrieval (encoding: cingulate gyrus, cuneus; retrieval: cingulate gyrus); a finding that confirms results from other studies investigating functional patterns during episodic/associative memory tasks (41–43). Accordingly, the few studies investigating the functional activation pattern in memory-related brain regions indicate disconnected (higher activated) brain regions within the default mode network (DMN) (44–47). The aberrant pattern in SZ patients in the DMN if associating faces to names may indicate an attentional deficit to focus on the relevant task and ignore irrelevant stimuli (41, 47). Nevertheless, the direction of the abnormal pattern within the DMN in SZ patients—reduced or increased activation—is yet to be investigated (41, 43, 47, 48). Other abnormal activations—encoding: middle frontal gyrus, middle and superior temporal gyrus, thalamus and occipital gyrus; retrieval: superior frontal gyrus and caudate—may be interpreted as compensatory mechanisms.

During ROI analyses, we observed significant group differences in left and right HC activation, driven during encoding by lower activation in HC bilaterally in patients compared to controls, and a continuum of activation pattern in the left HC, with the lowest activation in patients, followed by relatives and controls. During retrieval, lower activation was found in the HC bilaterally in patients compared to controls, and higher activation in the parahippocampal gyrus bilaterally. Controls showed higher activation in the left HC and lower activation in the right parahippocampal gyrus compared to relatives. This is in line with the meta-analysis by Achim and Lepage (2), as previously stated. They reported deactivated hippocampi during retrieval and increased activation of the parahippocampal gyrus. Activation in the HC may be related to the ability to build associations between faces and names (11). The hippocampus is involved in conscious recall whereas the parahippocampal gyrus is involved in familiarity with the recalled items (49). Previous studies suggested that SZ patients predominantly use familiarity with memorized items as strategy than consciously recall the items (2).

During ROI analyses, lower left PFC activation in SZ patients compared to controls and relatives during encoding, as well as lower PFC activation bilaterally during retrieval, in patients and relatives compared to controls was observed. There was no significant correlation between PFC activation with any clinical score across groups. Decreased activation within the PFC has been frequently reported in SZ (50). The PFC is known to be involved in the selection of items during recall (51); aberrant function during retrieval in SZ may indicate a failure in using efficient strategies (52, 53) leading to lower behavioral performance. This confirms the suggestion of a left-lateralized activation of the left PFC during encoding and a right-lateralized PFC activation during retrieval (9). Accordingly, Sperling et al. (11) reported a predominantly left-sided activation during the encoding of face-name pairs. Regarding our finding of mainly left-sided deactivation during encoding and bilateral deactivation during retrieval in SZ patients, this may be interpreted as a failure in normal left-lateralized encoding which may result in inferior task performance.

Regarding the activation pattern in the relatives group, we observed significant group contrasts in the right middle frontal gyrus, right superior parietal lobule, left lingual gyrus, left precuneus, left insula and in the right claustrum during encoding in contrast to controls. The observation of slight memory deficits, combined with minor functional abnormalities confirms the current knowledge from fMRI studies including first-degree relatives of SZ patients. For instance, Stolz et al. (18) reported the intermediate performance of relatives in episodic memory performance; they observed significant differences in the accuracy during retrieval exclusively. Accordingly, Skelley et al. (54) revealed deficits in first-degree relatives solely in verbal but not in visual episodic memory performance. This is in line with fMRI findings by Stolz et al. (18), who detected differences between relatives and controls during retrieval in the PFC, the thalamus and the insula (but not during encoding). Taken together, current knowledge leads to the assumption that relatives have subtle deficits in parts of the memory domain; underlined by minor fMRI differences; however, they may be able to compensate those alterations during certain conditions.

## Strength and Limitations

Regarding the patient sample, a widely discussed problem is the heterogeneity of symptoms and illness episodes in patients with psychotic disorders which may influence the results. We attempted to control for these potential characteristics and included only patients in a non-acute, stable condition and limited the patient sample to the paranoid-hallucinatory subtype. Furthermore, patients, first-degree relatives and healthy controls were well-matched regarding age, gender and years of education, which ensured a high level of comparability across groups in sociodemographic variables. Another important source of bias in studies with patients receiving pharmacological treatment is the potential influence of medication on functional imaging findings that has been discussed for SZ (55–58). Dazzan et al. (55) investigated how antipsychotic medication influences functional brain patterns based on typical antipsychotics (55), which may not be relevant for our patient sample (because they mainly received atypical antipsychotics). Other authors have discussed potential signal changes in frontal regions between unmedicated and medicated patients, as well as between patients receiving atypical vs. typical antipsychotics. The current knowledge indicates that antipsychotics may confound the functional activation pattern, and that atypical vs. typical medication might have different influence (55–58). However, most fMRI studies investigated medicated patients, and the authors attempt to solve this issue in controlling for equivalent doses of chlorpromazine. In our current study, we attempted to control these potential biases by only including patients who had been in a stable dosage for at least 4 weeks prior to testing. Furthermore, we computed medication equivalent doses according to the method of Wood (28) and performed correlation analyses to exclude potential associations between medication and imaging data. Moreover, none of the patients received benzodiazepines or tricyclic antidepressants at the time of testing. We also tested first-degree relatives who represent a medication-free sample and found several subtle changes that fit the findings of SZ patients. Furthermore, our results are congruent with findings from task-related fMRI studies, which increases confidence in the validity of our findings.

## Conclusions

Overall, the existing studies that investigate associative memory in SZ and SZ relatives showed inconsistent results. The number of studies that involved not only SZ patients but also their first-degree relatives is limited. Furthermore, only a few studies examined both—encoding and retrieval through behavioral and neuronal measurements. Therefore, we attempted to integrate several measures (behavioral, functional activation) and an additional subject group (unaffected first-degree relatives) into this study. To sum, we detected two major findings: the first one is that SZ patients have deficits in encoding and retrieval of face-name pairs; they have an expanded reaction time accompanied by lower performance. We assume that impairments in encoding and retrieval of face-name pairs are associated with deficient learning strategies (37, 38). This behavioral abnormality goes along with aberrant functional activation pattern during encoding and retrieval in SZ patients. As brain abnormalities were found in both task phases we suggest that there are deficits in both processes. The functional differences fit to other studies that observed deviant functional pattern in memory-relevant brain regions. The second major finding is that the group of unaffected SZ relatives showed only slightly differences in both, the functional activation as well as the behavioral performance.

The present results are important for biological models of schizophrenia that allow the investigation of high-risk samples and may thus aid a future biological classification of mental disorders. Accordingly, cognitive impairments influence the daily living of patients, being unfavorable for the outcome and are therefore a focus of current research. A better understanding of the underlying biological causes of persistent cognitive symptoms may help to develop specific therapeutic options, such as the functional remediation introduced by Martinez-Aran et al. (59) or the fMRI-based neurofeedback (60).

## Ethics Statement

This study was carried out in accordance with the recommendations of the ethical board of the medical department of Goethe University, Frankfurt/Main, Germany. The protocol was approved by the ethical board of the medical department of Goethe University, Frankfurt/Main, Germany. All subjects gave written informed consent in accordance with the Declaration of Helsinki.

## Author Contributions

VO, DP, RB, CK, and SM developed the study design. Data collection was performed by MS, DG, HS, and DK. Data analysis and interpretation were performed by VO and DK. VO, DK, and MS wrote the present article. AR, GA, and DL provided critical revisions. All the authors approved the final version of the manuscript for submission.

### Conflict of Interest Statement

The authors declare that the research was conducted in the absence of any commercial or financial relationships that could be construed as a potential conflict of interest.
