# Supplementary material for: Associative Memory Impairments Are Associated With Functional Alterations Within the Memory Network in Schizophrenia Patients and Their Unaffected First-Degree Relatives: An fMRI Study
Source: Front Psychiatry. 2019 Feb 19;10:33. doi: 10.3389/fpsyt.2019.00033 (PMC6391930; doi:10.3389/fpsyt.2019.00033)
Supplement: Supplementary file 1 [file Data_Sheet_1.docx]

**Supplementary material:**

**Table S1:** Clinical characteristics and psychiatric medication in the SZ patient group (n=27). Medications used were categorized as: *antipsychotics in monotherapy*, *antipsychotics in dualtherapy* (antipsychotics + antidepressants or + other antipsychotics). All SZ patients were treated with antipsychotic medication at time of measurement, however none of them received any benzodiazepines. Abbreviations: PANSS=positive and negative syndrome scale, PANSS pos=positive subscale, PANSS neg=negative subscale, PANSS gen=general subscale, PANSS total=total score, M=mean score, SD=standard deviation.

|  | **SZ patients (M [SD]** |
| --- | --- |
| **Duration of illness** *(years)* | 10.87 (7.73) |
| **Age of onset** *(years)* | 27.22 (7.56) |
| **PANSS** | pos: 16.82 (4.95), neg: 16.28 (5.88), gen: 32.32 (7.29), total: 65.42 (15.15) |
| **Medication categories** | antipsychotics monotherapy  antipsychotics dualtherapy |
| **Substances** | Risperidon (n=8)  Clozapin (n=3)  Quetiapin (n=4) |
|  | Risperidon + Aripiprazol (n=1)  Risperidon + Flupentixol (n=2)  Olanzapin + Aripiprazol (n=4)  Clozapin + Aripiprazol (n=3)  Haloperidol + Quetiapin (n=2) |
| **Chlorpromazine equivalent**  *(mg /day)* | 367 mg/day (12.34mg/day) |

**Description of the face-name association task (originated by Sperling et al., 2002):**

Stimuli consisted of 30 grey-scaled photographs of forward-facing, neutral faces drawn from AR face database (Martinez and Benavente, 1998). Furthermore, 120 popular first names were chosen from a German online name database (www.beliebtevornamen.de). 30 names were randomly paired with faces during encoding and retrieval runs, while 90 names were used as distractors during retrieval only. Face–name pairs were alternated across all runs and each retrieval run was designed to test memory for face–name pairs encoded in the previous run. All subjects performed three encoding and retrieval runs - one run lasted approximately four minutes. During encoding runs, 30 face – name pairs were presented for eight seconds each. The interstimulus interval (ITI) consisted of a checkerboard pattern. Its duration varied from 8 to 12 s in order to “jitter” trials onset times and thereby minimize multicollinearity of event-related fMRI analyses. During retrieval the previously presented face–name pairs were shown with three distractor names in a pseudorandomized order. Subjects were instructed to indicate the correct name via button press. During retrieval, each face with four names was presented for eight seconds.

**Detailed information about the imaging sequences for this experiment:**

For T1 weighted structural imaging, an optimized 3D modified driven equilibrium fourier transform sequence (3D MDEFT; (Deichmann et al., 2004) with following parameters was applied: acquisition matrix 256 x 256, repetition time (TR)=7.92ms, echo time (TE)=2.48ms, field of view (FOV)=256mm, 176 slices, and 1.0 mm slice thickness. During the acquisition of three functional runs (T2* weighted Echo-Planer-Imaging (EPI) sequence (64x64, TR=2000 ms, TE=30 ms, FOV=192 mm, flip angle=90°, 121 images), a face–name association paradigm originated by Sperling et al. (2002) was presented.

**Preprocessing pipeline of the functional runs:**

We synchronized the stimulus presentation with the fMRI sequence at the beginning of each trial using MRI pulses that triggered the ongoing presentation of stimuli in the Presentation® software. For MRI data, a standard preprocessing pipeline was applied. To permit T1 equilibration effects, the first two images per functional run were excluded. We pre-processed and analyzed the fMRI data using BrainVoyager QX software (Brain Innovation, Maastricht, The Netherlands). We applied the following pre-processing steps: slice-time correction, motion correction, linear trend removal and highpass temporal filtering of 2 cycles per time course. Co-registration of the functional data to the anatomical scans was performed with automated scripts in BrainVoyager QX, following by a manual control of the anatomical alignment. We transformed the 3D anatomical scans into Talairach space (Talairach & Tournoux, 1988) using a 12-point affine transformation as implemented in the BrainVoyager software, and subsequently used the parameters of this transformation to transform the coregistered functional data. We then resampled the 3D functional data set to a voxel size of 1x1x1 mm.

**Table S2:** Results of the self-constructed questionnaire to explore memory strategies.

*Abbreviations*: names=low utterance of the names, visualization=visualization, recollection=recollection (because) of striking features, relation=relating of the names through a story, association=association of the faces/names with known persons, other=any other (answer yes/no).

| **Strategy** | **SZ** | **REL** | **CON** | **Significance** |
| --- | --- | --- | --- | --- |
| **usage in total** *yes (%)* | 75.91 | 79.12 | 78.43 | *χ*^2^=0.43, *p*=0.51 |
| **names** *yes (%)* | 44.82 | 43.54 | 49.23 | *χ*^2^=0.63, *p*=0.43 |
| **visualization** *yes (%)* | 70.37 | 72.31 | 69.23 | *χ*²=0.57, *p*=0.44 |
| **recollection** *yes (%)* | 89.74 | 85.43 | 87.34 | *χ*^2^=0.422, *p*=0.52 |
| **relation** *yes (%)* | 30.35 | 32.12 | 29.56 | *Χ*^2^=1.31, *p*=0.25 |
| **association** *yes (%)* | 51.72 | 50.12 | 49.21 | *χ*^2^=0.70, *p*=0.81 |
| **other** *yes (%)* | 66.75 | 68.12 | 64.12 | *χ*^2^=1.58, *p*=0.20 |
| **attention** *(points)* | 3.12 (0.43) | 3.51 (0.94) | 3.54 (0.43) | *Z*=1.14, *p*=0.41 |
| **concentration** *(points)* | 3.87 (1.42) | 3.98 (0.57) | 4.01 (0.65) | *Z*=2.36 *p*=0.25 |

**Table S3:** Group comparisons of region-of-interest-analyses (ROI) regarding the hippocampus (HC) and prefrontal gyrus (PFC) for encoding and retrieval. *Abbreviations:* SZ=schizophrenia patients, REL=schizophrenia relatives, REL=first-degree relatives, CON=controls. PFC=prefrontal cortex, HC=hippocampus, L=left, R=right, BA=Broadman area. *Note*: * =no Brodmann area; *p*<0.05, corrected using cluster thresholding approach; Talairach coordinates, anatomical regions and Brodmann areas refer to peak voxel of cluster.

| Anatomical region | R/L | BA | Talairach coordinates | | | Cluster Size | *t*_(76)_ |
| --- | --- | --- | --- | --- | --- | --- | --- |
|  |  |  | x | y | z | (voxels/mm³) |  |
|  | **encoding** | | | | | | |
| Parahippocampal Gyrus | L  R | *  * | -11  17 | -39  -32 | 1  1 | 2495  5055 | *t*=5.89, *p*<0.001  *t*=4.28, *p*<0.005 |
| HC | L  R | *  * | -27  27 | -9  -13 | -15  -11 | 3034  4844 | *t*=6.24, *p*<0.001  *t*=6.01, *p*<0.001 |
| PFC | L |  | -43 | 19 | 24 | 5175 | *t*=7.01, *p*<0.001 |
| **SZ<CON** |  |  |  |  |  |  |  |
| HC | L | * | -33 | -8 | -14 | 151 |  |
|  | R | * | 35 | -2 | 14 | 130 |  |
| PFC | L | * | -47 | 29 | 22 | 195 |  |
| **REL<REL** |  |  |  |  |  |  |  |
| HC | L | * | -34 | -9 | -13 | 168 |  |
| **SZ<REL** |  |  |  |  |  |  |  |
| HC | L | * | -36 | -28 | -15 | 105 |  |
| PFC | L | * | -36 | 14 | 23 | 114 |  |
|  | **retrieval** | | | | | | |
| Parahippocampal Gyrus | L  R | *  * | -13  15 | -40  -31 | -3  -3 | 2267  3500 | *t*=4.71, *p*<0.001  *t*=5.67, *p*<0.001 |
| HC | L  R | *  * | -21  20 | -22  -29 | -4  -3 | 1774  3594 | *t*=4.90, *p*<0.001  *t*=6.51, *p*<0.001 |
| PFC | L | * | -42 | 19 | 25 | 3721 | *t*=6,12, *p*<0.001 |
|  | R | * | 42 | 8 | 32 | 3725 | *t*=5.14, *p*<0.001 |
| **SZ<CON** |  |  |  |  |  |  |  |
| HC | L  R  R  L | *  *  *  * | -28  35  27  -20 | -29  -19  -33  -2 | -2  -10  8  -11 | 1524  229  401  192 |  |
| PFC | L | * | -37 | 18 | 22 | 589 |  |
|  | R | * | 34 | 21 | 25 | 789 |  |
| **SZ>CON** |  |  |  |  |  |  |  |
| Parahippocampal Gyrus | L  R | *  * | -12  14 | -30  -31 | -13  -11 | 180  103 |  |
| **REL<CON** |  |  |  |  |  |  |  |
| HC | L | * | -35 | -25 | -8 | 281 |  |
| PFC | L | * | -50 | 8 | 28 | 153 |  |
|  | R | * | 33 | 20 | 27 | 160 |  |
| **REL>CON** |  |  |  |  |  |  |  |
| Parahippocampal Gyrus | R | * | 14 | -21 | -13 | 47 |  |
| **SZ<REL** |  |  |  |  |  |  |  |
| HC | L  R | *  * | -20  13 | -36  -35 | 8  11 | 1321  939 |  |
| **SZ>REL** |  |  |  |  |  |  |  |
| HC | L | * | -10 | -29 | -12 | 170 |  |
|  |  |  |  |  |  |  |  |

Figure S1: Illustration of the ROIs masks for ROI analyses of hippocampus (HC) (y=-31) and prefrontal cortex (PFC) (y=10). The anatomically defined regions were based on the automated anatomical labeling atlas in WFU PickAtlas v2.0 (Tzourio-Mazoyer et al., 2002). *Cluster*: HC: -26, -23, -7 [11345 voxels]; 29, -19, -7 [12312], PFC: -40, 20, 22 [3583); 41, 10, 33 (3393).
