# Supplementary figures and images for: Associative Memory Impairments Are Associated With Functional Alterations Within the Memory Network in Schizophrenia Patients and Their Unaffected First-Degree Relatives: An fMRI Study
Source: Front Psychiatry. 2019 Feb 19;10:33. doi: 10.3389/fpsyt.2019.00033 (PMC6391930; doi:10.3389/fpsyt.2019.00033)

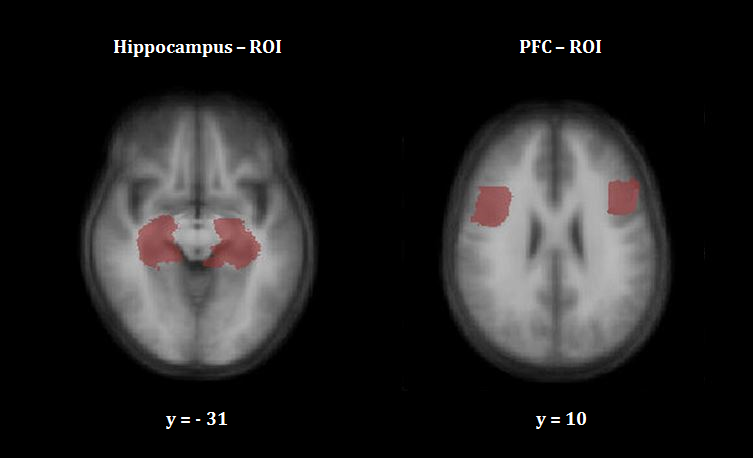

Supplement: Supplementary file 2 [file Image_1.TIF]
